# Modulation of TTX-sensitive voltage-dependent Na^+ ^channels by β-bungarotoxin in rat cerebellar neurons

**DOI:** 10.1186/1471-2202-13-36

**Published:** 2012-03-29

**Authors:** Da Guo, Wei Xiang, Angela Seebahn, Cord-Michael Becker, Olaf Strauß

**Affiliations:** 1 Experimental Ophthalmology, Eye Hospital, University Medical Center Regensburg, Franz-Josef-Strauss Allee 11, 93053 Regensburg, Germany; 2 Institute of Biochemistry, Emil-Fischer Zentrum, Friedrich-Alexander University Erlangen-Nürnberg, Fahrstraße 17, 91054 Erlangen, Germany

## Abstract

**Background:**

The modulation of voltage-dependent Na^+ ^channels by lipid metabolites such as arachidonic acid or eicosanoids plays a role in physiological functions as well as in degenerative diseases. So far TTX-resistant channels were found mainly to be regulated by lipid metabolites.

**Results:**

We investigated the lipid-dependent modulation of TTX-sensitive (TTX-s) Na^+ ^channels using β-bungarotoxin (β-BuTX, 10 pM), which has an intrinsic phospholipase-A2 activity, and indomethacin (10 μM), which blocks cyclooxygenase activity in primary cerebellar neurons. To investigate TTX-s Na^+ ^channels, whole-currents were measured under K^+^-free conditions and blocked by 10 nM TTX. The currents resulting from calculating the difference of currents measured in the presence and the absence of TTX were used for further analysis. Application of indomethacin mainly changed the current kinetics but has only minor effects on voltage-dependence. In contrast β-BuTX increased the maximal current amplitude and shifted the voltage-dependent activation towards more negative potentials. The effects of β-BuTX were blocked by indomethacin. Analysis of lipid metabolites which accumulate by treatment with β-BuTX using MALDI-TOF MS showed an increase of cyclooxygenase reaction products in relation to arachidonic acid.

**Conclusions:**

In summary, we conclude that TTX-sensitive Na^+ ^channels can be directly modulated by cyclooxygenase reaction products leading to higher activity at less depolarized potentials and subsequent higher excitability of neurons. Since activation of cyclooxygenase is also involved in pathways leading to apoptotic cells death this could play a role in degenerative diseases of the CNS and highlights a possible protective effect of cyclooxygenase inhibition.

## Background

Na^+ ^channels are known to be modulated by lipid metabolites, such as arachidonic acid or prostaglandins. These modulations serve physiological functions such as neurotransmitter-dependent change in excitability, pain generation or inflammation-dependent hyperalgesia [1-7]. Also in degenerative processes the modulation of Na^+ ^channels by prostaglandins plays a role. In cerebral ischemia, neuronal cell death can be caused by excitotoxicity, which is based on strong depolarization and over-excitation of neurons [1,2,6,8]. The increase in Na^+ ^channel activity by lipid metabolites might further contribute to the over-excitation.

Both tetrodotoxin-sensitive (TTX-sensitive or TTX-s) Na^+ ^channels and TTX-resistant (TTX-r) Na^+ ^channels are modulated by either arachidonic acid or eicosanoids [5,9]. In contrast to TTX-s Na^+ ^channels, the interaction of TTX-r Na^+ ^channels and eicosanoids has been so far well characterized. The increase of TTX-r Na^+ ^channel activity by prostaglandins is known to be involved in hyperalgesia [2,4]. However, the effects of prostaglandins on Na^+ ^channel currents were found to be rather mediated by G-proteins. Direct application of arachidonic acid reduces Na^+ ^channel activity by changing the maximal current amplitude, influencing Na^+ ^current inactivation kinetics and shifting the voltage-dependent activation [5,10,11]. These effects seem to be one of the major mechanisms of dopamine-induced decrease in neurotransmitter release and reduction of Na^+^-dependent action potentials [10]. The effects of arachidonic acid could be blocked by cyclooxygenase inhibitor indomethacin [5]. Thus the effects of arachidonic acid seem to be more mediated by the metabolites of arachidonic acid.

Lipid metabolites are also known to contribute to apoptotic cell death in several degenerative diseases including ischemia [1,2,12,13]. In order to study mechanisms leading to apoptosis, animal toxins were often used. In this respect, the application of β-bungarotoxin (β-BuTX) is one of the most established models [14-21]. β-BuTX is a component of the venom of the Taiwanese banded krait *Bungarus muticinctus*. The toxic effect of β-BuTX is related to its intrinsic phospholipase-A2 activity [14,15]. The mechanisms triggering neuronal cell death via β-BuTX include the increase in intracellular free Ca^2+^, increased Ca^2+ ^influx through NMDA-receptors, caspase-3 activation, production of reactive oxygen species and NO production, as well as K^+ ^channel inhibition [16-20,22-27].

The modulation of Na^+ ^channels by lipid metabolites serves many functions but was analyzed only in detail for TTX-r Na^+ ^channels. The effects on TTX-s Na^+ ^channels were so far less investigated and the effects of β-BuTX as a model for apoptotic cell death had not been investigated on the activity of Na^+ ^channels, although Na^+ ^channel modulation by lipids could play a role in over-excitation. Therefore, the aim of the study was to investigate the effects of the modulators of lipid metabolism indomethacin and β-BuTX on TTX-s Na^+ ^channels in rat cerebellar neurons. For this purpose, TTX-sensitive whole-cell currents were measured from primary cerebellar granule neurons (CGN) under exposure to β-BuTX and indomethacin. Matrix-assisted laser-desorption-ionization time-of-flight mass spectrometry (MALDI-TOF MS) was used to determine changes in lipid metabolites.

## Results

**Figure 1:**
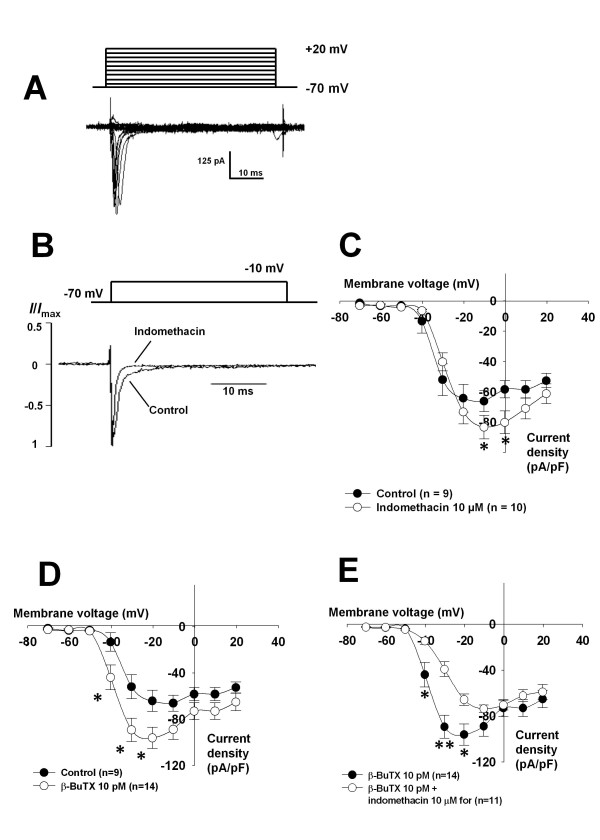
**Effects of indomethacin and β-bungarotoxin on TTX-sensitive currents in cerebellar neurons**. **1A: **Example of TTX-sensitive currents: currents were activated by depolarization of cells from a holding potential of -70 mV in nine voltage-steps with 10 mV increasing amplitude and 50 ms duration; currents were measured in the presence and in the absence of TTX (10 nM). Currents measured in before application of TTX were subtracted from currents measured in the presence of TTX and used for further analysis. **1B: **Effect of indomethacin (10 μM): currents were stimulated by a voltage-step from -70 mV to -10 mV and plotted as relative current to the maximal current amplitude. Indomethacin led to a faster time-dependent activation. **1C: **Effect of indomethacin on TTX-sensitive Na^+ ^currents: current density was plotted against the potentials of the electrical stimulation. Indomethcin led to increased current density. **1D: **Effect of β-Bungarotoxin (β-BuTX, 10 pM) on TTX-sensitive currents. Currents were normalized to the membrane capacitance and plotted as current density against the potentials of the electrical stimulation. **1E: **Inhibition of β-BuTX effects by indomethacin: currents were measured in the presence of either β-BuTX or in the presence of β-BuTX together with indomethacin; current density was calculated and plotted against the potentials of the electrical stimulation. (* p < 0.05).

Under K^+^-free conditions, depolarization of CGN cells from a holding potential of -70 mV led to the activation of fast activating and inactivating inward currents. These currents activated at potentials more positive than -40 mV which peaked at -10 mV. Application of tetrodotoxin (TTX; 10 nM) completely blocked these currents. Thus these currents could be identified as currents through neuronal voltage-dependent Na^+ ^channels. Under these conditions the TTX-sensitive current was 90.0 ± 1.2% (n = 7). In order to analyze the effects of β-BuTX on pure TTX-sensitive currents, in all cells of the experiments shown here, we used only the currents which resulted from subtraction of the currents measured before TTX application from those currents measured in the presence of TTX in the same cell (Figure 1A; Additional file 1: Figure S1). Thus only the currents which were blocked by TTX were further analyzed and TTX-resistant currents had no influence on data analysis.

In order to study the influence of eicosanoids on Na^+ ^channel activity, two substances were used. Indomethacin blocks cyclooxygenase which catalyzes the first step in the production of different eicosanoids from arachidonic acid. β-BuTX is known to show intrinsic phospholipase-A2 activity which catalyzes the production of arachidonic acid. Incubation of cells with indomethacin (10 μM) mainly led to an acceleration of the time-dependent activation. In the presence of cyclooxygenase blocker the current activated much faster by a voltage-step from -70 mV to -10 mV (Figure 1B). Furthermore, at the voltages between -10 and 0 mV indomethacin increased the maximal current density of Na^+ ^channel currents (Figure 1C). On the other hand, incubation with β-bungarotoxin (β-BuTX; 10 pM) increased the current density and led to a shift in the voltage-dependence towards more negative values (Figure 1D). When cells were incubated with β-BuTX together with indomethacin the TTXs Na^+ ^channel current amplitudes were smaller and voltage-dependence was shifted towards more positive values compared to the currents measured in the presence of β-BuTX alone (Figure 1E). Thus inhibition of cyclooxygenase by indomethacin was able to block the effects of β-BuTX. β-BuTX seems to favorably affect Na^+ ^channels. Voltage-dependent Ca^2+ ^channels showed changes neither in current density nor in voltage-dependence in the presence of 10 pM β-BuTX (Additional file 2: Figure S2).

**Figure 2:**
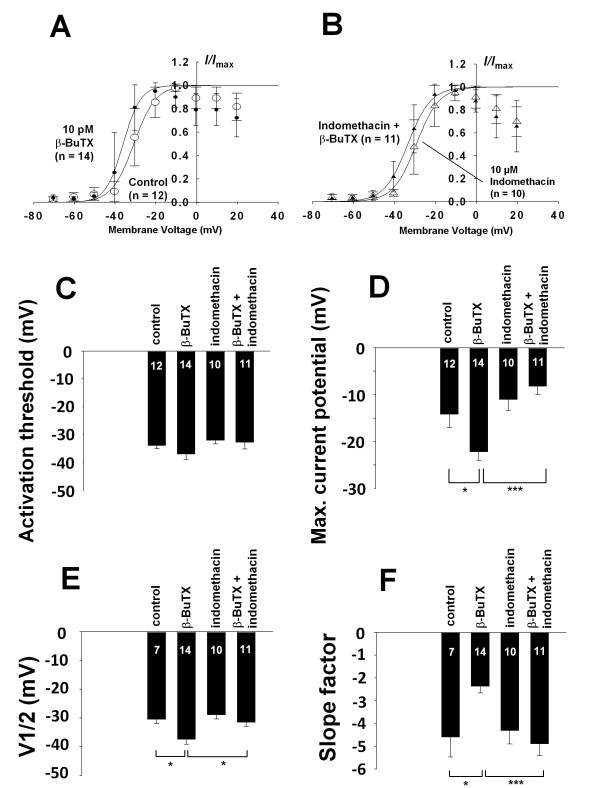
**Analysis of changes in voltage-dependence of TTX-sensitive currents by indomethacin and β-bungarotoxin**. **2A: **Effect of β-BuTX on voltage-dependence by current/voltage-relationships: currents were normalized to the maximal current amplitude and plotted against the potentials of the electrical stimulation. Curves were fitted using the Boltzmann equation. In the presence of β-BuTX the voltage-dependent activation was shifted towards more negative potentials. **2B: **Effect of indomethacin and β-BuTX on voltage-dependence by current/voltage-relationships: relative currents were plotted against the membrane potentials; curves were fitted using the Boltzmann equation. Indomethacin reversed the effect of β-BuTX. **2C: **Comparison of activation thresholds from the current voltage-relationships: neither β-BuTX nor indomethacin showed any effect on activation threshold. **2D: **Comparison of the potentials of maximal current amplitude obtained from current/voltage-relationships. In the presence of β-BuTX the maximal current amplitude was observed at much more negative potentials. **2E: **Comparison of potentials of half maximal activation obtained from Boltzmann fits; β-BuTX shifted the voltage-dependent activation towards more negative voltages which was reversed by indomethacin. **2F: **Comparison of the slope-factors of current/voltage-relationships obtained from Boltzmann fits; with β-BuTX the curves were much steeper than under control conditions which could be reversed by indomethacin. (*p < 0.05; **p < 0.01; ***p < 0.001).

In order to compare the effects of indomethacin and β-BuTX on voltage-dependence of Na^+ ^channel currents normalized current/voltage plots were fitted using the Boltzmann equation and fitting parameters were compared under each experimental condition (Figure 2A and 2B). The activation threshold was not changed by either indomethacin or β-BuTX (Figure 2C). However, the voltage of maximal current amplitude was shifted towards more negative membrane potentials by β-BuTX, which could be blocked by indomethacin, whereas indomethacin alone had no significant effect on the voltage of maximal current amplitude (Figure 2D). The Boltzmann analysis of voltage-dependence revealed that the change in the voltage of maximal current amplitude was due to a shift of the voltage of half maximal activation towards more negative voltages in the presence of β-BuTx (Figure 2E). Again, this effect could be blocked by the application of indomethacin, while indomethacin itself had no effect on the voltage of maximal activation. Furthermore, β-BuTX significantly reduced the slope factor of the current/voltage curve indicating a remarkable increase in current amplitude with depolarizing membrane voltages (Figure 2F). This effect was blocked by indomethacin whereas indomethacin alone had no effect on the slope factor. Thus β-BuTX shifted the voltage-dependence of Na^+ ^channels towards more negative membrane voltages leading to an increase in Na^+ ^currents to larger current amplitudes with depolarizing voltages.

**Figure 3:**
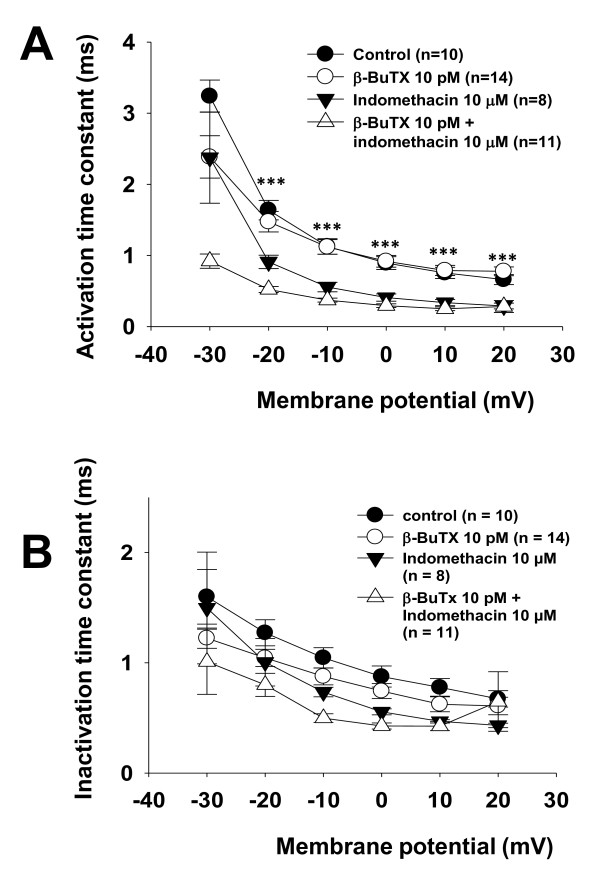
**Analysis of changes in the time-dependent activation of TTX-sensitive currents by β-BuTX or indomethacin**. **3A: **Comparison of the activation time constants: activation time constants were estimated by single exponential fits for each voltage. Indomethacin led to acceleration of the time-dependent activation which could not reversed by β-BuTX. **3B: **Comparison of the inactivation time constants: inactivation time constants were estimated by single exponential fits and plotted against the membrane potentials. Neither β-BuTX nor indomethacin showed any influence on time-dependent inactivation. (* *p *< 0.05; **p < 0.01; ***p < 0.001).

As shown in Figure 1 indomethacin and β-BuTX had also effects on the activation kinetics of the Na^+ ^currents. For a thorough analysis of the Na^+ ^kinetics we plotted the activation time constants and the inactivation time constants against the test voltages of the stimulation protocol (Figure 3A). The activation time constants and the inactivation time constants were estimated using a single exponential fit of the current curve during activation and inactivation respectively. Here we found that indomethacin led to faster time-dependent activation than under control conditions which could not be reversed by β-BuTX which alone has no effect on the time-dependent activation (Figure 3A). On the other hand, either indomethacin or β-BuTX had no effects on the time-dependent inactivation (Figure 3B).

**Figure 4:**
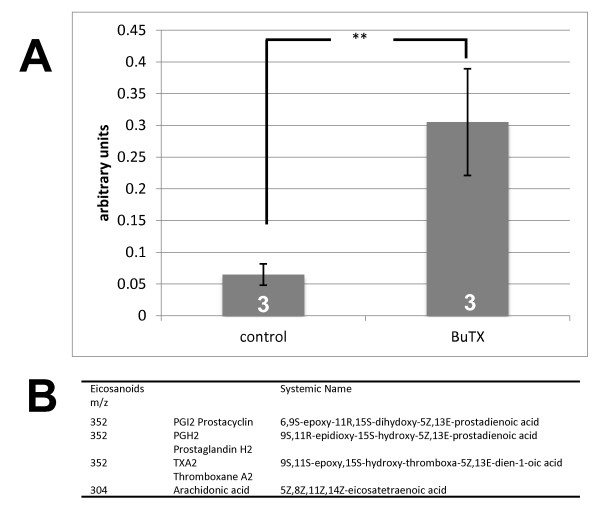
**Analysis of the ratio of arachidonic acid and metabolites of arachidonic acid in neurons under influence of β-bungarotoxin**. **4A: **Ratio of relative signal intensities of arachidonic acid metabolites to arachidonic acid was calculated for each individual spectrum after MALDI-TOF analysis. 4**B: **Table of mass signals of different eicosanoids in the MALDI-TOF spectrum. (** *p *< 0.01).

β-BuTX has an intrinsic phospholipase-A2 activity which may lead to an accumulation of intracellular arachidonic acid or increased formation of metabolic products of arachidonic acid, catalyzed e.g. by cyclooxygenase or lipoxygenase. Since indomethacin, a cyclooxygenase inhibitor reverses the β-BuTX-induced increase in Na^+ ^channel currents it is likely that the β-BuTX -effect is mediated by cyclooxygenase reaction products. Thus we examined the reaction products of cyclooxygenase which arose from treatment of cells with β-BuTX using MALDI-TOF MS techniques (Figure 4). The semi quantitative analysis of the reaction products concentrated on cyclooxygenase products from arachidonic acid and determined as ratio between reaction products and arachidonic acid. Arachidonic acid was measured at m/z 304 whereas the reaction products were measured at m/z 352. As a result we found that treatment with β-BuTX increased the amount of these reaction products in relation to the arachidonic acid concentration. Thus the major effect of β-BuTX on arachidonic acid metabolism was not an accumulation of arachidonic acid, but an increase of the level of its reaction products prostacyclin, prostaglandin and/or thromboxane. It should be mentioned that this method did not enable us to measure absolute concentrations.

## Discussion

In this paper we analyzed the effects of reaction products of lipid metabolism on Na^+ ^channel activity in neurons. For this purpose two substances that change the lipid metabolism were used: indomethacin, which inhibits cyclooxygenase and β-BuTX a peptide from snake venom with intrinsic phospholipase-A activity. We could show that Na^+ ^channel activity is sensitive to cyclooxygenase reaction products of arachidonic acid. These reaction products enhanced the Na^+ ^activity by increasing the maximal current amplitude and shifts of the voltage-dependent activation towards more negative potentials.

In order to analyze the activity of voltage-dependent Na^+ ^channels depolarization-induced whole-cell currents from cerebellar neurons were measured in the presence and absence of TTX under K^+^-free conditions in all cells. After subtraction of the currents, which were measured in the presence of TTX from those measured in the absence of TTX, the currents blocked by TTX were calculated. Further data analysis was only done using the subtracted currents. Since we used 10 nM TTX to inhibit voltage-dependent Na^+ ^channel currents, only TTX-s Na^+ ^channels were investigated [28]. These TTX-sensitive currents were used for further analysis of Na^+ ^channel activity under influence of lipid metabolites.

As a first result we found that after inhibition of cyclooxygenase activity the time-dependent activation was faster and increased the maximal current amplitude at depolarized potentials than under control conditions. This could be either due to an accumulation of substrates of cyclooxygenase or a decrease of its reaction products. The main substrate of cyclooxygenase reaction is arachidonic acid. Thus the effects of cyclooxygenase inhibition are possibly due to an increase in arachidonic acid level. Studies which examined the effects of arachidonic acid on Na^+ ^channel activity could show that extracellular application of arachidonic acid alone has an inhibitory effect. Extracellular application of arachidonic acid led to faster time-dependent inactivation, shifts in the voltage-dependence and smaller Na^+ ^current amplitude in dorsal root ganglion cells and in striatal neurons [5,10]. TTX-r Na^+ ^channels in cardiac cells also showed a reduced current amplitude and voltage-dependence as a direct effect of arachidonic acid [11]. The effects of arachidonic acid were found to be caused partly by arachidonic acid itself and partly by arachidonic metabolites [5,11]. However, a recent paper showed that intracellular application of arachidonic acid leads to an increase in the Na^+ ^channel amplitude. Since we observed a higher maximal current and a faster time-dependent activation Na^+ ^channels, the effect of cyclooxygenase inhibition is rather due to an increase of intracellular arachidonic acid concentration [29]. It should be noted that indomethacin is a non-selective blocker which cannot differentiate between cyclooxygenase-1 and -2 [30,31]. The effects of indomethacin alone were observed in un-stimulated cells and could be more due to inhibition of cyclooxygenase-1.

For the first time, we investigated the effects of β-BuTX on the Na^+ ^currents in cerebellar neurons. Interestingly, we found that treatment with β-BuTX led to no changes in the current kinetics but to an increase in the maximal current amplitude and to a shift of the voltage-dependent activation towards more negative potentials. Thus β-BuTX treatment led to the activation of Na^+ ^channels at negative potentials and led to larger current amplitudes. In summary, β-BuTX effects would lead to an increase of excitability of cerebellar neurons. In general, the increase in excitability would correspond to a toxic action of β-BuTX by inducing neurotoxicity which is based on over-excitation of the neurons involving NMDA receptors [14,16,20,22,27,32]. In addition to that, by its phospolipase activity β-BuTX leads to apoptosis. Here, as a second effect, the products of phospholipase activity directly promote the signaling cascade leading to apoptosis [14-16,19,22,32]. Then the modulation of Na^+ ^channels to a higher activity would be a synergistic effect enhancing neurotoxicity.

90% of the whole-cell currents were TTX-sensitive currents. The remaining TTX-resistant currents resembled voltage-dependent Ca^2+ ^channel currents. Na^+ ^channels and Ca^2+ ^channels are closely related in their structures. Thus we tested whether β-BuTX also influenced ion channels which are accounting for the TTX-resistant part of the whole-cell currents under K^+^-conditions. Interestingly β-BuTX did not change the activity of voltage-dependent Ca^2+ ^channels. Taking into account the analysis method and the fact under our recording conditions only Na^+ ^channels were affected by β-BuTX we conclude that activation of lipid metabolism by β-BuTX favorably modulates TTX-s Na^+ ^channels. However, a possible influence on other ion channels such as K^+ ^channels, Cl channels or ionotropic neurotransmitter receptors cannot be excluded.

The effects of β-BuTX were inhibited by treatment with indomethacin, indicating that the inhibition of cyclooxygenase reverses the effects of β-BuTX. This observation suggests the involvement of lipid molecules in β-BuTX-dependent increase in Na^+ ^channel activity. The β-BuTX phospholipase activity leads to the production of arachidonic acid. Arachidonic acid can be metabolized by lipoxygenase and by cyclooxygenase. Since indomethacin can block the effects of β-BuTX, products of the cyclooxygenase pathway are responsible for the modulation of Na^+ ^channel activity. In this way indomethacin cannot be regarded as an antagonist of the β-BuTX. This hypothesis is supported by comparing the effects of indomethacin and β-BuTX on Na^+ ^channel current amplitude. Both led to an increase in current amplitude but indomethacin at voltages between -10 and 0 mV and β-BuTX at voltages between -40 and -20 mV. Furthermore, indomethacin accelerated the time-dependent activation whereas β-BuTX had no effects on current kinetics. Thus, both effects possibly result from different metabolites. The effect from indomethacin possibly results from arachidonic acid and the effect of β-BuTX results from cyclooxygenase reaction products. Moreover, this hypothesis is further supported by our observations from MALDI-TOF MS analysis of lipid metabolites arising from incubation with β-BuTX. Since cyclooxygenase metabolizes arachidonic acid and the effects of β-BuTX were blocked by cyclooxygenase inhibitor we performed a relative quantification of the cyclooxygenase products versus arachidonic acid. As a result we found that β-BuTX led to an increase of the cyclooxygenase products in relation to arachidonic acid. Arachidonic acid is a reaction product of phospholipase-A which can be metabolized by lipoxygenase or by cyclooxygenase. Although MALDI-TOF MS analysis enabled us only measure relative amount and no absolute concentrations our finding, however, suggests that a considerable amount of arachidonic acid is quickly metabolized by cyclooxygenase. Thus, incubation of cerebellar neurons with β-BuTX directly leads to an increase of cyclooxygenase reaction products. Thus, in summary, the β-BuTX effects on Na^+ ^channel activity are then due to cyclooxygenase reaction products.

Application of β-BuTX to neurons was used as a model to investigate cellular mechanisms related to apoptosis during neurotoxicity. The effects of β-BuTX on Na^+ ^channel activity could be interpreted as an increase in excitability of the neurons which also represents a basic mechanism in neurotoxicity. This is supported by the knowledge that cyclooxygenase products are involved in promoting cell death in neurotoxicity. Since indomethacin could prevent the effects of β-BuTX, indomethacin may have a beneficial effect on neurotoxicity in general. Cyclooxygenase inhibition is used in early treatment of stroke, possibly this treatment has more beneficial effects than currently known. This study provides experimental evidence for such a mechanism by which indomethacin prevents cyclooxygenase reaction products dependent increase in excitability of neurons.

## Conclusions

In summary we conclude that β-BuTX enhances the activity of TTX-sensitive Na^+ ^channels in cerebellar neurons. Since cyclooxygenase inhibition reversed the effect of β-BuTX was due to cyclooxygenase reaction products. Thus the inhibition of cyclooxygenase could reduce excitability of neurons acting at the Na^+ ^channel side and therefore beneficial to reduce neurotoxicity.

## Methods

### Preparation of primary neuronal culture

Rat CGNs were prepared from 1-4 days old BDE (black hooded) rats. Cerebelli were isolated from rats and stored in PBS on ice. After centrifugation (3 min, 800 rpm), PBS was discarded. Subsequently cerebellar granule cells were dissociated by 0.01% papain in the presence of 0.1% Dispase II and 0.01% DNase for 30 min. The tissue was triturated gently with glass pipettes twice for dissociation. Dissociated cells were pelleted by centrifugation (800 rpm for 3 min), and were washed twice by DMEM/Han's F12 medium with 10% FCS gold, 1% penicillin/streptomycin. 70 μm cell strainers were used to remove undigested tissue. Cells were re-suspended in DMEM/Ham's F12 medium with 10% FCS gold, 1% penicillin/streptomycin, and were plated on poly-L-lysine-coated glass coverslips in 24-well culture plates. Cultures were maintained at 37°C in a humidified atmosphere with 5% CO_2_. One day after plating, culture medium was changed to SFM (serum free), in which FCS gold was replaced by 2% B-27 supplement. Five micromolar cytosine arabinoside was also added to the culture medium to arrest the growth of non-neuronal cells. All experiments were performed using CGNs cultured at least for 7 days. All cell culture media, nutrients and supplements were purchased from PAA (Pasching, Austria).

### Whole-cell recordings of Na^+ ^channels currents

Cells attached to a coverslip were transferred into a recording chamber on the stage of an inverted microscope. Ionic currents were recorded under voltage-clamp conditions by the perforated whole-cell patch clamp technique using nystatin as ionophor. Glass pipettes were pulled from borosilicate glass tubes (GB150T-8P, Science Products, Germany) to give a resistance of 3-4.5 MΩ using DMZ Universal Puller (Zeitz Ausgburg Germany). The external solution contained 130 mM NaCl, 5 mM CsCl, 4 mM MgCl_2_, 2 mM CaCl_2_, 10 mM HEPES and 5 mM Glucose at pH 7.3. The internal solution contained 140 mM CsCl, 2 mM MgCl_2_, 1 mM CaCl_2_, 2.5 mM EGTA, 10 mM HEPES, adjusted to pH 7.3. Recordings were performed at room temperature and cells were held at -70 mV. Whole cell currents were acquired with an EPC 7 amplifier (HEKA electronics Inc., Germany) and TIDA5.22 (HEKA electronics Inc., Germany) software. Tetrodotoxin (Alomone Labs, Ltd., Jerusalem, Israel) was dissolved in Millipore water as 3.23 mM stock solution and stored at -20°C. Tetrodotoxin was diluted in external solution to 10 nM before the experiment. Nystatin (Sigma-Aldrich Chemie Gmbh, Munich, Germany) used for perforated patch was dissolved in DMSO as 25 mg/ml stock solution, which were stored at 4°C for 1 day. Nystatin can be used for 3 hours after diluted in internal solution to 175 μg/ml. Currents through voltage-dependent Ca^2+ ^channels were measured using the same solutions with extracellular 10 mM Ba^2+ ^as a charge carrier and 10 nM TTX to block Na^+ ^channels. The currents were activated using the same electrical stimulation as for the measurement of Na^+ ^channel currents.

### Application of β-bungarotoxin and indomethacin

β-bungarotoxin and indomethacin (Sigma-Aldrich Chemie GmbH, Munich, Germany) were solved in water and directly applied to the extracellular solution. According to Tseng et al. maximal effects on membrane permeability for Ca^2+ ^without leading to cell death was observed after 2 h [25]. Therefore, cells were incubated for 2 h in either extracellular solution alone, extracellular solution with β-bungarotoxin (10 pM), indomethacin (10 μM) or both substances for 2 h prior to the measurements of Na^+ ^channel currents.

### MALDI-TOF MS analysis of arachidonic acid and its metabolites

After treatment, cells were immediately frozen in liquid nitrogen and stored at -80°C. After thawing, tissue samples were homogenized and sonicated. Lipids were subjected to classical chloroform/methanol extraction [33], transferred to a clean tube, and dried in a vacuum centrifuge and subsequently resolved (2% w/v) in chloroform. For MALDI-TOF MS, samples were mixed with matrix consisting of 0.5 M 2,5-dihydroxybenzoic acid in methanol with 0.1% trifluoroacetic acid [34]. Aliquots were spotted on a steel target allowed to dry at ambient temperature and subjected to mass spectrometrical analysis. Mass spectra were obtained using an Autoflex MALDI-TOF MS (Bruker Daltonics, Bremen, Germany) equipped with a nitrogen laser (λ = 337 nm). Mass spectra were acquired in reflectron and positive ion mode. Each spectrum consists of the average of 100 laser shots.

For the semi-quantitative analysis of arachidonic acid and its metabolites prostacyclin I2, prostaglandin H2 and thromboxane A2, the relative intensities of mass signals [M + H^+^] were determined and added up. Obtained m/z values were compared to the lipid database LIPID MAPS http://www.lipidmaps.org/. The ratio of relative signal intensities of arachidonic acid metabolites to arachidonic acid was calculated for each individual spectrum, and averaged over three individual samples.

### Data analysis

Patch clamp data were analyzed by TIDA 5.22 (HEKA electronics Inc., Germany) software and SigmaPlot (Jandel Scientific, San Rafael, CA) software. To analyze voltage-dependence of channel activation, the current-voltage relationships were fitted according to Boltzmann equation.

$$I={\left[\text{1}\;\text{ + }\;\text{exp}\;\left(V_{0.\text{5}}-V\right)/k\right]}^{-\text{1}}$$

*I *is current through ion channels, *V*_0.5 _is the potential at which *I *is half-maximum, *V *is test potential, and *k *is the slope factor of the Boltzmann term. The accuracy of fit was determined by coefficient of determination, *R*^2^.

The time course of current inactivation was fitted according to an exponential function.

$$I\left(t\right)=a\times \text{exp}\left(-t/\tau \right)+c$$

*I*(*t*) is the current at time *t *after the peak of sodium current; a is the coefficient of activation; *τ *is the inactivation time constant; and c is the maximal current. Activation time of current was measured in TIDA 5.22. The time at which peak current appeared was subtracted by the beginning of stimulation. Threshold and the potential which evoked peak current were analyzed by using raw data.

### Statistics

All data are presented as mean ± SEM and "n" refers the number of experiments. Current/voltage-relationships were plotted and fitted using the Boltzmann equation for each individual cell. Values of each individual Boltzmann fit were used for statistical analysis. Statistical significance was determined by *P < 0.05 *(*), *P < 0.01 *(**), and *P < 0.001 *(***) using student's *t*-test.

## Competing interests

The authors declare that they have no competing interests.

## Authors' contributions

DG has performed the patch-clamp experiments and data analysis. WX contributed to the β-BuTX and the MALDI-TOF experiments and the coordination of the study. AS has performed the MALDI-TOF experiments, data analysis and proof-reading of the manuscript. C-MB planned and designed the experiments on lipid metabolism and MALDI-TOF analysis. OS wrote the manuscript, planed and supervised the electrophysiology experiments and organized the study between Erlangen and Regensburg. The manuscript was read, corrected and approved by all authors.

## Supplementary Material

Additional file 1 **Figure S1 Isolation of TTX-sensitive currents. A: **Electrical stimulation: The cells were electrically stimulated by 10 depolarizing voltage-steps of 50 ms duration with 10 mV increasing amplitude from a holding potential of -70 mV. **B: **Whole-cell currents measured under K^+^-free conditions: Voltage-dependent currents elicited by the electrical stimulation shown in A. **C: **Voltage-dependent currents in the presence of 10 nM TTX: Whole-cell currents from the same cell as shown B. **D: **TTX-sensitive currents resulting from subtraction of the currents shown in B and C. Click here for file

Additional file 2 **Figure S2 Effects of β-BuTX on voltage-dependent Ca^2+ ^channels. A: **Currents through voltage-dependent Ca^2+ ^channels were measured under extra- and intracellular K^+^-free conditions in the presence of 10 mM Ba^2+ ^as a charge carrier and 10 nM TTX to block Na^+ ^channel currents. The cells were electrically stimulated by 10 depolarizing voltage-steps of 50 ms duration with 10 mV increasing amplitude from a holding potential of -70 mV. **B: **In the presence of Ba^2+ ^the electrical stimulation results in fast activating and slowly inactivating inward currents. **C: **Current/voltage plot showing the effects of 10 pM β-BuTX on Ba^2+ ^currents. The currents activated at -40 mV and reached maximal current amplitude at 0 mV. The currents were inhibited by 20% after application of 10 μM (+)BayK8644 indicting that several types of high-voltage activated Ca^2+ ^channels contribute to the Ba^2+ ^currents. Application of β-BuTX did change neither current density nor voltage-dependence of the Ba^2+ ^currents. Click here for file
