# Supplementary material for: Modulation of TTX-sensitive voltage-dependent Na+ channels by β-bungarotoxin in rat cerebellar neurons
Source: BMC Neurosci. 2012 Mar 29;13:36. doi: 10.1186/1471-2202-13-36 (PMC3338087; doi:10.1186/1471-2202-13-36)
Supplement: Additional file 1 — Figure S1 Isolation of TTX-sensitive currents. A: Electrical stimulation: The cells were electrically stimulated by 10 depolarizing voltage-steps of 50 ms duration with 10 mV increasing amplitude from a holding potential of -70 mV. B: Whole-cell currents measured under K+-free conditions: Voltage-dependent currents elicited by the electrical stimulation shown in A. C: Voltage-dependent currents in the presence of 10 nM TTX: Whole-cell currents from the same cell as shown B. D: TTX-sensitive currents resulting from subtraction of the currents shown in B and C. [file 1471-2202-13-36-S1.PDF]

Additional File: Fig. 1

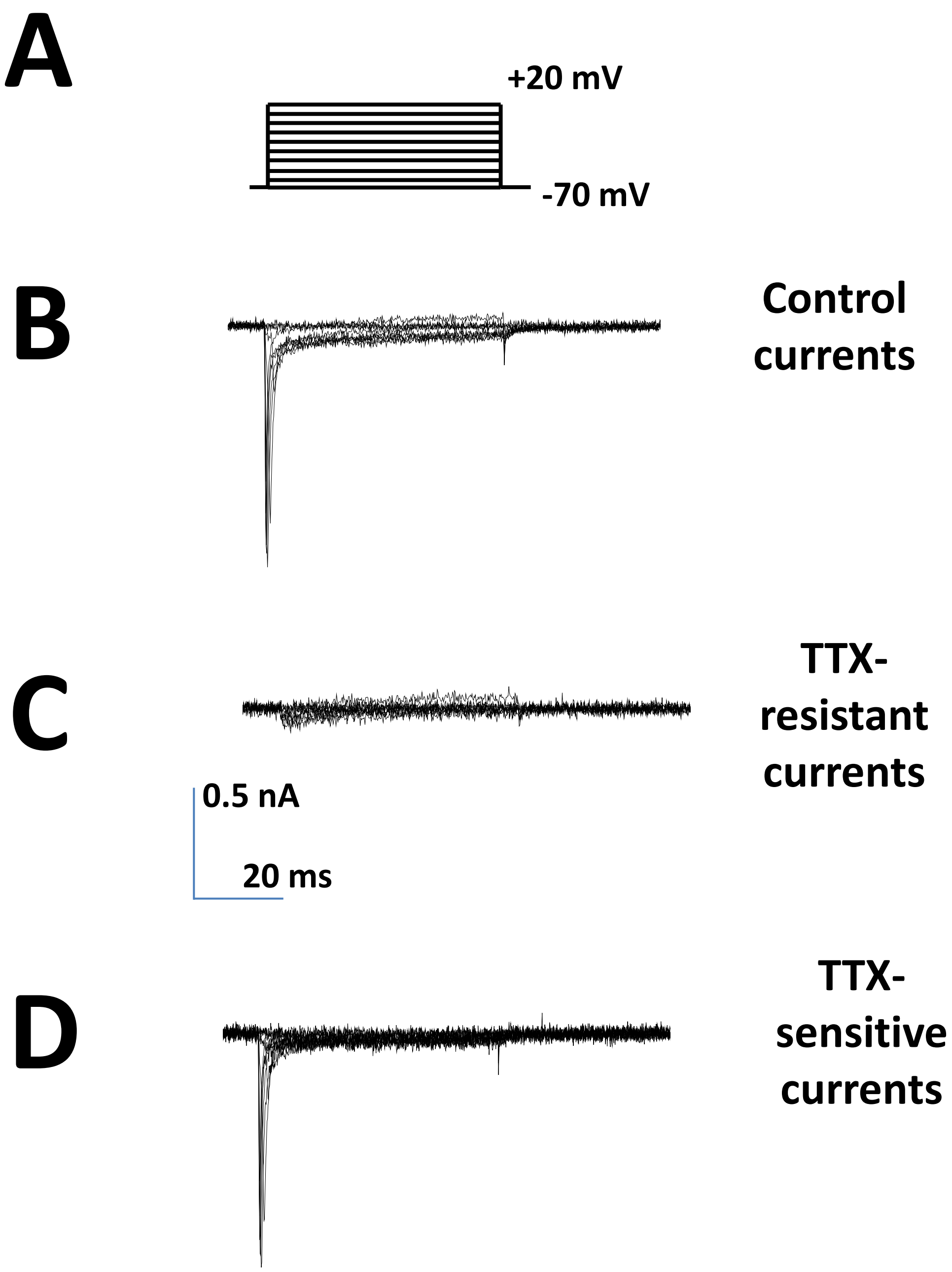

Additional Files Figure 1: Isolation of TTX-sensitive currents

A: Electrical stimulation: The cells were electrically stimulated by 10 depolarizing voltage-steps of 50 ms duration with 10 mV increasing amplitude from a holding potential of -70 mV.

B: Whole-cell currents measured under K^+-free conditions: Voltage-dependent currents elicited by the electrical stimulation shown in A.

C: Voltage-dependent currents in the presence of 10 nM TTX: Whole-cell currents from the same cell as shown B.

D: TTX-sensitive currents resulting from subtraction of the currents shown in B and C
