# Supplementary material for: Modulation of TTX-sensitive voltage-dependent Na+ channels by β-bungarotoxin in rat cerebellar neurons
Source: BMC Neurosci. 2012 Mar 29;13:36. doi: 10.1186/1471-2202-13-36 (PMC3338087; doi:10.1186/1471-2202-13-36)
Supplement: Additional file 2 — Figure S2 Effects of β-BuTX on voltage-dependent Ca2+ channels. A: Currents through voltage-dependent Ca2+ channels were measured under extra- and intracellular K+-free conditions in the presence of 10 mM Ba2+ as a charge carrier and 10 nM TTX to block Na+ channel currents. The cells were electrically stimulated by 10 depolarizing voltage-steps of 50 ms duration with 10 mV increasing amplitude from a holding potential of -70 mV. B: In the presence of Ba2+ the electrical stimulation results in fast activating and slowly inactivating inward currents. C: Current/voltage plot showing the effects of 10 pM β-BuTX on Ba2+ currents. The currents activated at -40 mV and reached maximal current amplitude at 0 mV. The currents were inhibited by 20% after application of 10 μM (+)BayK8644 indicting that several types of high-voltage activated Ca2+ channels contribute to the Ba2+ currents. Application of β-BuTX did change neither current density nor voltage-dependence of the Ba2+ currents. [file 1471-2202-13-36-S2.PDF]

Additional File: Fig. 2

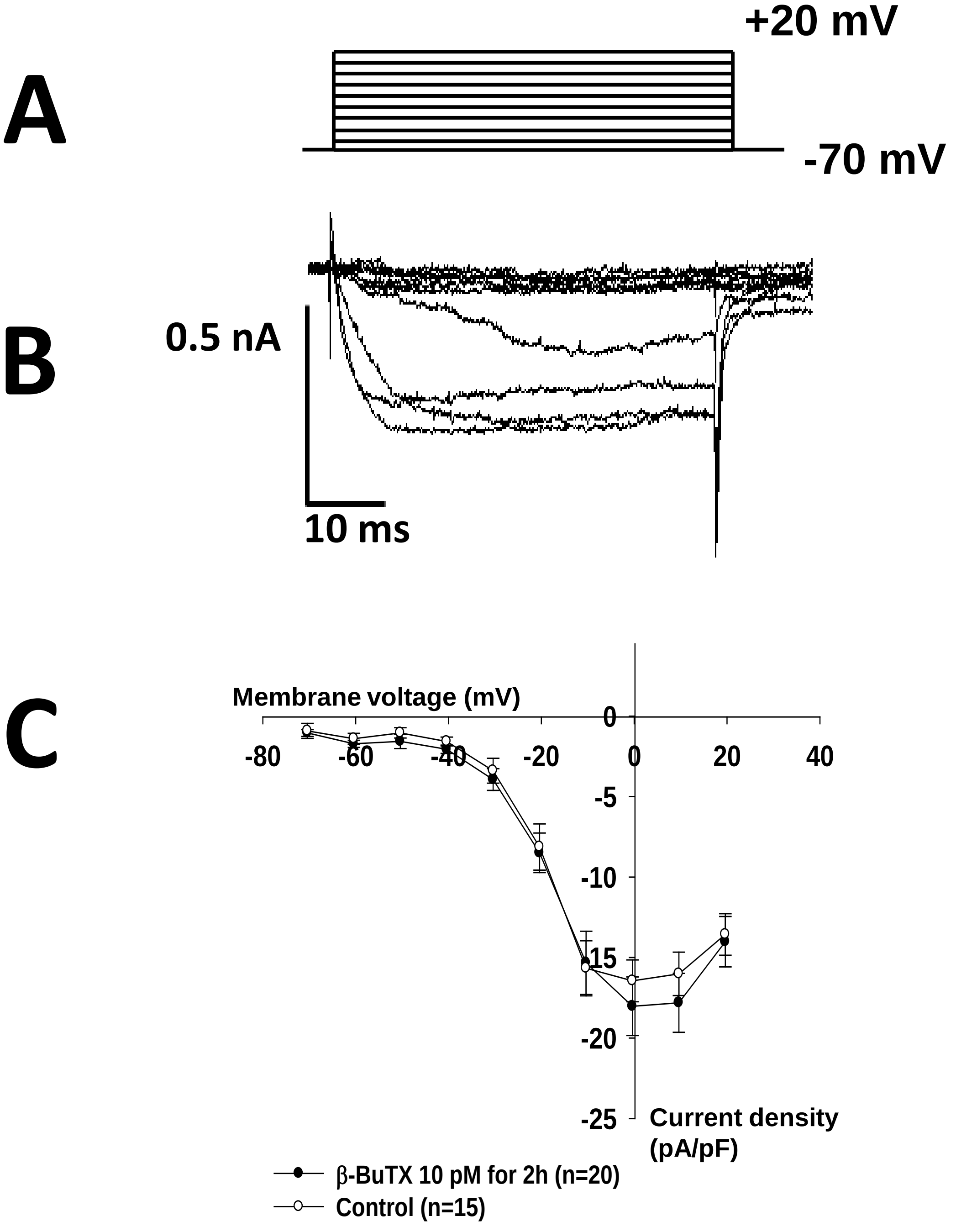

Additional Files Figure 2: Effects of β-BuTX on voltage-dependent Ca^{2+} channels

A: Currents through voltage-dependent Ca^{2+} channels were measured under extra- and intracellular K^{+}-free conditions in the presence of 10 mM Ba^{2+} as a charge carrier and 10 nM TTX to block Na^{+} channel currents. The cells were electrically stimulated by 10 depolarizing voltage-steps of 50 ms duration with 10 mV increasing amplitude from a holding potential of -70 mV.

B: In the presence of Ba^{2+} the electrical stimulation results in fast activating and slowly inactivating inward currents.

C: Current/voltage plot showing the effects of 10 pM β-BuTX on Ba^{2+} currents. The currents activated at -40 mV and reached maximal current amplitude at 0 mV. The currents were inhibited by 20 % after application of 10 µM (+)BayK8644 indicting that several types of high-voltage activated Ca^{2+} channels contribute to the Ba^{2+} currents. Application of β-BuTX did change neither current density nor voltage-dependence of the Ba^{2+} currents.
